# Genome-Wide Identification and Characterization of Melon *bHLH* Transcription Factors in Regulation of Fruit Development

**DOI:** 10.3390/plants10122721

**Published:** 2021-12-10

**Authors:** Chao Tan, Huilei Qiao, Ming Ma, Xue Wang, Yunyun Tian, Selinge Bai, Agula Hasi

**Affiliations:** 1 Key Laboratory of Herbage & Endemic Crop Biology, Ministry of Education, School of Life Sciences, Inner Mongolia University, Hohhot 010070, China; 1120200475@mail.nankai.edu.cn (C.T.); huohuli-1982@163.com (H.Q.); 13804714146@sina.cn (M.M.); yunyuntian412@hotmail.com (Y.T.); 2 Department of Plant Biology and Ecology, College of Life Sciences, Nankai University, Tianjin 300071, China; atinysnow@163.com; 3 Medical College, Inner Mongolia MINZU University, Tongliao 028000, China

**Keywords:** *bHLH*, fruit ripening, *CmbHLH32*, melon

## Abstract

The *basic helix-loop-helix* (*bHLH*) transcription factor family is one of the largest transcription factor families in plants and plays crucial roles in plant development. Melon is an important horticultural plant as well as an attractive model plant for studying fruit ripening. However, the *bHLH* gene family of melon has not yet been identified, and its functions in fruit growth and ripening are seldom researched. In this study, 118 *bHLH* genes were identified in the melon genome. These *CmbHLH* genes were unevenly distributed on chromosomes 1 to 12, and five *CmbHLHs* were tandem repeat on chromosomes 4 and 8. There were 13 intron distribution patterns among the *CmbHLH* genes. Phylogenetic analysis illustrated that these CmbHLHs could be classified into 16 subfamilies. Expression patterns of the *CmbHLH* genes were studied using transcriptome data. Tissue specific expression of the *CmbHLH32* gene was analysed by quantitative RT-PCR. The results showed that the *CmbHLH32* gene was highly expressed in female flower and early developmental stage fruit. Transgenic melon lines overexpressing *CmbHLH32* were generated, and overexpression of *CmbHLH32* resulted in early fruit ripening compared to wild type. The *CmbHLH* transcription factor family was identified and analysed for the first time in melon, and overexpression of *CmbHLH32* affected the ripening time of melon fruit. These findings laid a foundation for further study on the role of bHLH family members in the growth and development of melon.

## 1. Background

Transcription factors (TFs) play important roles in regulating plant growth, development, stress response and signal transduction [1,2,3,4]. Basic helix-loop-helix (bHLH) TFs is one of the largest TF superfamilies in plant [5]. Studies on the *bHLH* gene family in various species will increase our understanding of their evolution and functions. So far, comprehensive identification of the *bHLH* gene family has been performed in a range of plant species, such as *Arabidopsis*, *Brachypodium distachyon*, *Solanum lycopersicum*, *Arachis hypogae*, and *Malus pumila* [6,7,8,9,10]. Plant *bHLH* genes are characterized by a basic helix-loop-helix domain which is highly conserved in evolution [11]. The bHLH domain contains 50–60 amino acids and can be separated into two regions: the region at the N-terminal end is a DNA binding domain, comprised of approximately 13–17 amino acids [5]; the C-terminal end is an HLH domain, containing two amphipathic α-helices connected by a loop region with variable length, which helps to form a dimerization domain, and allows the formation of homo or heterodimeric complexes [12]. With regard to the bHLH protein domain, 19 amino acids are conserved and functional for DNA binding or dimerization formation. A highly conserved HER motif (His 5-Glu 9-Arg 13) is considered important for binding to specific DNA sequences; however, some atypical *bHLH* genes lack the N-terminal binding region which may have different mechanisms in plant development [13].

Phylogenic analysis of 544 plant bHLHs shows that plant bHLH proteins form 26 distinct subfamilies, and these subfamilies are highly conserved throughout plant evolution; among them, 20 subfamilies were present in early land plants 443 Ma [14]. However, plant bHLH subfamilies have discernible phylogenetic relationships, but it is still unknown whether plant *bHLH* genes evolved from a common ancestor [5].

Plant bHLH proteins are involved in a wide range of biological processes and participate in the regulation of plant growth and development, abiotic/biotic stress response, hormone signaling, iron homeostasis and secondary metabolism [15,16,17,18,19,20,21]. Nevertheless, research on *bHLH* gene regulation in fruit growth and development is limited. Fruit growth is triggered by the fertilization of the ovule, with drastic changes, including cell division and expansion, secondary metabolite accumulation, and carbohydrate biosynthesis, which involve transcription and regulation of many genes [22,23]. For the climacteric fruit such as melon, the ripening process also accompanies an increase in ethylene emission and a burst of respiratory climacteric [24]. Recent research on tomato reveals that overexpressing *SlbHLH22* results in early flowering, accelerated fruit ripening, accumulation of carotenoids by activation of the *SlSFT* or *SlLFY* genes, and exogenous ACC, IAA, ABA, and ethephon would upregulate the expression of *SlbHLH22* [25].

Melon (*Cucumis mleo* L.) is an important horticultural crop worldwide. In 2017, the worldwide production of melon was more than 49 million tons, and China produced over one-third (FAO). Melon is also an attractive model plant of the Cucurbitaceae family for studying fruit development and ripening, especially for respiratory climacteric fruit [26]. Although *bHLH* genes have been suggested to be involved in a wide range of metabolic, physiological, and developmental processes in plants, very few studies focus on *bHLH* genes in the regulation on fruit ripening. In this study, we identified the melon *bHLH* gene family members, and analysed their expression patterns during melon fruit development using high-throughput sequencing data. Transgenic lines overexpressing *CmbHLH32* was generated to study the effect on fruit ripening, and yeast two-hybrid analysis was used to research the transcriptional activation activity of *CmbHLH32*. Our findings shed light on the molecular properties and evolution patterns of the *CmbHLH* gene family and demonstrate the biological function of the *CmbHLH32* gene in melon fruit growth and ripening.

## 2. Results

### 2.1. Identification of CmbHLH Proteins and Conserved Domain Alignment

A total of 169 CmbHLH candidate protein sequences were obtained by Hidden Markov Model (HMM) analysis. Using the blast method, 213 protein sequences were found, then repetitive sequences were removed. The remaining sequences were searched against the CmbHLH proteins of the PlantTFDB database. After that 214 sequences were reserved and submitted to CDD domain search; then 159 sequences were found with a bHLH conserved domain above the minimum domain hit, and the redundant sequences of the 159 proteins were removed. Finally, 118 sequences remained as *CmbHLHs* gene models for the foyther analysis and renamed based on their chromosome localization (Table S1).

These putative CmbHLH lengths varied from 84 to 707 aa, the molecular weight ranged from 9.48 kDa to 75.97 kDa, and the theoretical isoelectric points (PI) ranged from 4.52 to 10.27 (Table S1). The *CmbHLH* gene density (0.442) was lower than that in *Cucumis sativus* (0.527), which also belongs to Cucurbitaceae. The reason may be the smaller genome size of *Cucumis sativus* (203.0 Mb) compared to *Cucumis melo* (364.0 Mb) [27]. Additionally, the CmbHLH density was slightly higher than that in *Citrus sinensis* (0.420), which has a similar genome size (*Citrus sinensis*, 367.0 Mb).

**Figure 1:**
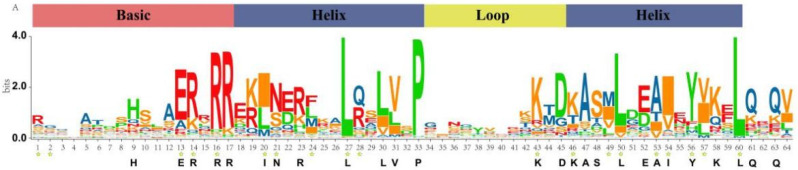
Sequence logo of bHLH domain of CmbHLH. The high letter represents the conservation of the sequence at that site. Stars represent the conservation sites of bHLH in *Arabidopsis*. Bold letters below represent the conservation sites of the bHLH domain that have a consensus ratio higher than 50%.

Multiple sequence alignment of the bHLH domain of CmbHLH proteins shows that 24 amino acid residues in their bHLH domains were conserved with a consensus ratio greater than 50%. The bHLH domain is highly conserved and comprises two functionally distinct regions. The basic region of the bHLH domain determines the DNA-binding activity of target genes, Figure 1 shows the bHLH domain logo of CmbHLH (Figure 1). The basic region of CmbHLH proteins contains 5 conserved amino acids, and HLH has 19 conserved amino acids. Previously described a classification of bHLH proteins that classified the bHLH proteins into four groups A, B, C and D. The classification was based on DNA-binding specificity as well as conservation of amino acids at certain positions [13]. According to the criterion, 8 CmbHLH were classified into the A group, 69 were located in the B group, and 16 and 33 CmbHLHs belonged to the C and D groups, respectively.

**Figure 2:**
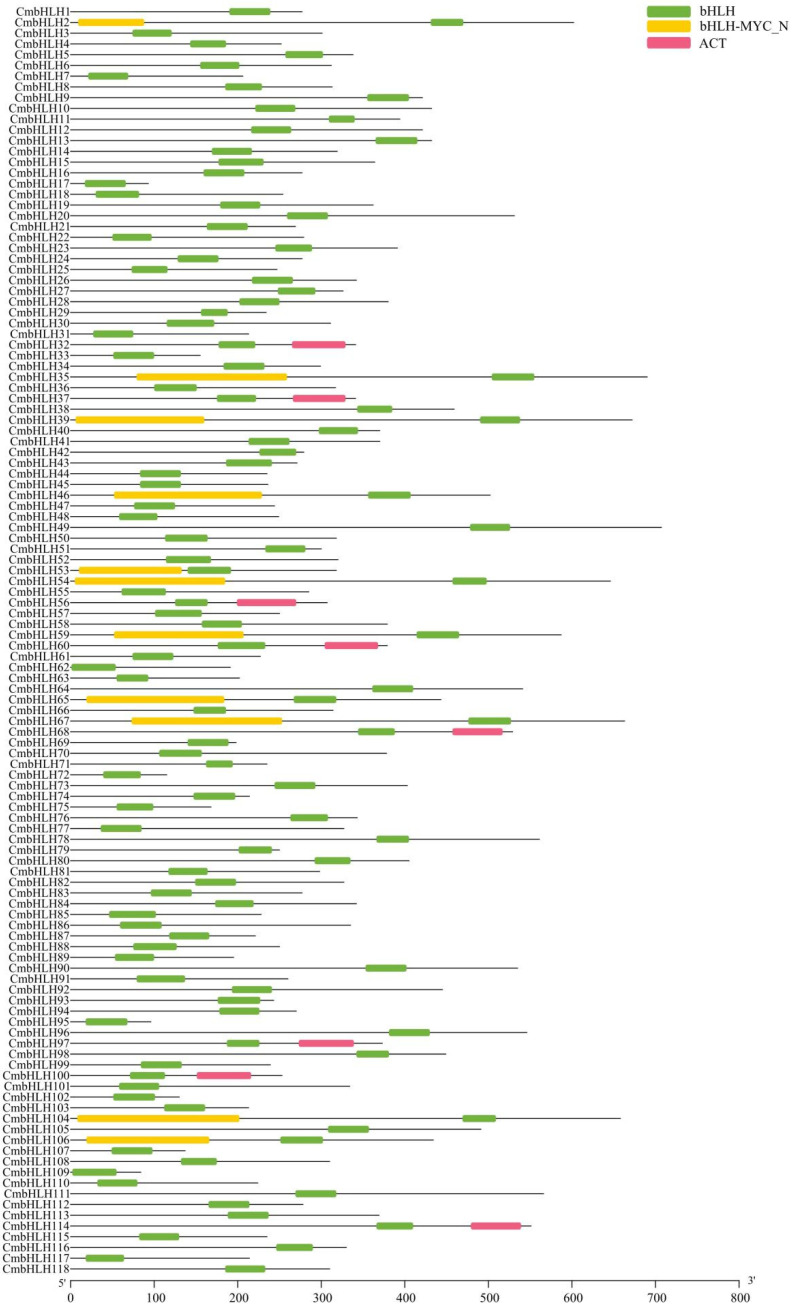
The domain distribution and types of CmbHLH.

Domain analysis of CmbHLHs illustrated that two kinds of domains were found in CmbHLHs in addition to the bHLH domain (Figure 2). One domain is bHLH_MYC_N, found in 11 CmbHLH. All of the CmbHLH bHLH_MYC_N domains were located in the N-terminus of the proteins, and bHLH domains in the C-terminus of the proteins. Apart from the bHLH and bHLH_MYC_N domains, the ACT (Aspartokinase, Chorismate, and TyrA) domain was also identified in eight CmbHLH (CmbHLH32, CmbHLH37, CmbHLH56, CmbHLH60, CmbHLH68, CmbHLH97, CmbHLH100, CmbHLH114), and all of the ACT domains were located in the C terminal of the bHLH domain.

### 2.2. Phylogenetic, Motif Analysis and Gene Structure of Cmbhlh

**Figure 3:**
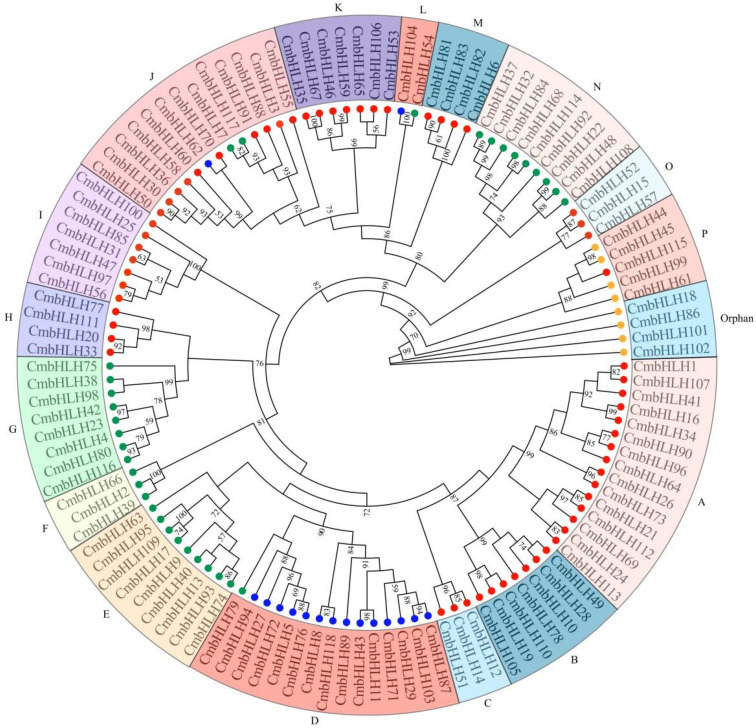
ML phylogenetic tree of CmbHLHs with predicted DNA-binding activities. The tree shows the 16 phylogenetic subfamilies marked with different colored backgrounds. The different colored dots indicate four bHLH DNA binding activties groups, A (yellow), B (red), C (blue) and D (green), according to Atchley (2003).

To evaluate the evolutionary relationships of CmbHLHs, we conducted a phylogenetic analysis based on full-length protein sequences. Applying the Maximum likelihood (ML) method, we assigned the *CmbHLH* genes into 16 subfamilies and 4 orphan genes (Figure 3). Subfamilies A, D and J were the largest groups; the smallest subfamily (L) had only 2 members. According to the phylogenetic tree, the *CmbHLH* binding activities were phylogenetically clustered, which was consistent with the previously report [28]. For example, nine subfamily members (A, B, C, H, I, J, K, M and O) belonged to the B group protein, and four subfamily members (E, F, G and M) were classified into the group D protein.

**Figure 4:**
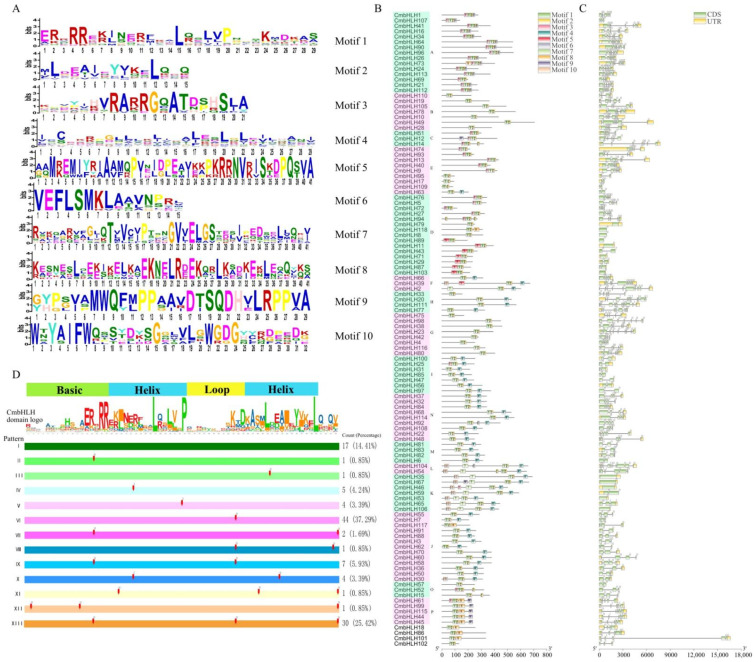
Sequence logo and arrangement of conserved motifs, gene structure and intron pattern of *CmbHLH*. (**A**) Sequence logo of 10 conserved motifs of CmbHLH. (**B**) Arrengement of conserved motifs. Motifs labeled with 1 to 10 in different colores. (**C**) Intron-exon structure of CmbHLH. The exon, intron and UTR are represented by green rectangle, lines and yellow rectangle, respectively; the numbers (0–3) represent the intron phase. (**D**) Intron distribution pattern of bHLH of CmbHLH. The bars (labeled from I to XIII) illustrate the intron distribution pattern of the coding sequence of the bHLH domain within CmbHLHs. The arrows indicate the intron positions, and the numbers (0–3) above the arrows represent the intron phase. The count and percentage of genes in each pattern are shown on the right.

The evolutionary relationships of these CmbHLH proteins were also determined by conserved motifs. A total of 10 conserved motifs were characterized from CmbHLH proteins (Figure 4A,B). Among these motifs, motif 1 and 2 were annotated to the bHLH domain (IPR011598, IPR036638), and motif 7 and 10 were annotated to the transcription factor bHLH-MYC-N-terminal (IPR025610). The subfamily L contains the highest number of motifs (six motifs). CmbHLH14 and CmbHLH94 possess two and three motif-2 respectively. The motif distribution and construction pattern exhibited similar models within subfamilies.

The exon-intron organizations of CmbHLHs were examined to gain more insight into the evolution of the bHLH family of melon. The exon number of CmbHLHs varied from 1 to 11 (Figure 4C), whereas the exon-intron organizations were phylogenetically related. For example, the CmbHLHs with one exon were clustered in two subfamilies (D and K). All the members of subfamily I have two exons. Intron distribution analysis of bHLH domain within all CmbHLH proteins exhibit 13 intron distribution patterns, and this pattern was strongly related to the subfamilies of CmbHLH (Figure 4D). As shown in Figure 4, 85% of CmbHLH have intron insertion in their bHLH domain sequence region. Although the intron positions and lengths were varied, only five intron insertion positions of CmbHLH were unconserved. Overall, the conserved motif arrangement and composition and the gene structure of CmbHLH genes, together with the phylogenetic analysis results, could strongly support the reliability of the classification.

### 2.3. Chromosomal Distribution and Collinearity Analysis of CmbHLH

**Figure 5:**
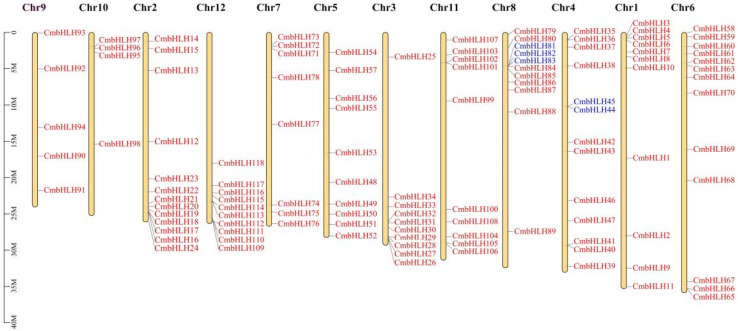
Chromosomal distribution and tandem repeat gene of *CmbHLH*. Blue characters indicate tandem repeat genes.

*CmbHLH* genes were distributed unevenly among twelve chromosomes of melon (Figure 5). However, the distribution of *CmbHLH* genes did not show either a chromosome length correlation or a phylogenetic correlation. Gene tandem duplication may be involved in gene family enlargement and maintenance of gene copy numbers. Thus, we analyzed the tandem duplication events of *CmbHLH*. Five genes were confirmed to be tandemly duplicated genes. Three of them (*CmbHLH81*, *CmbHLH82* and *CmbHLH83*) located on chromosome 8, and two of tandem duplicated genes (*CmbHLH45* and *CmbHLH44*) were located on chromosome 4.

**Figure 6:**
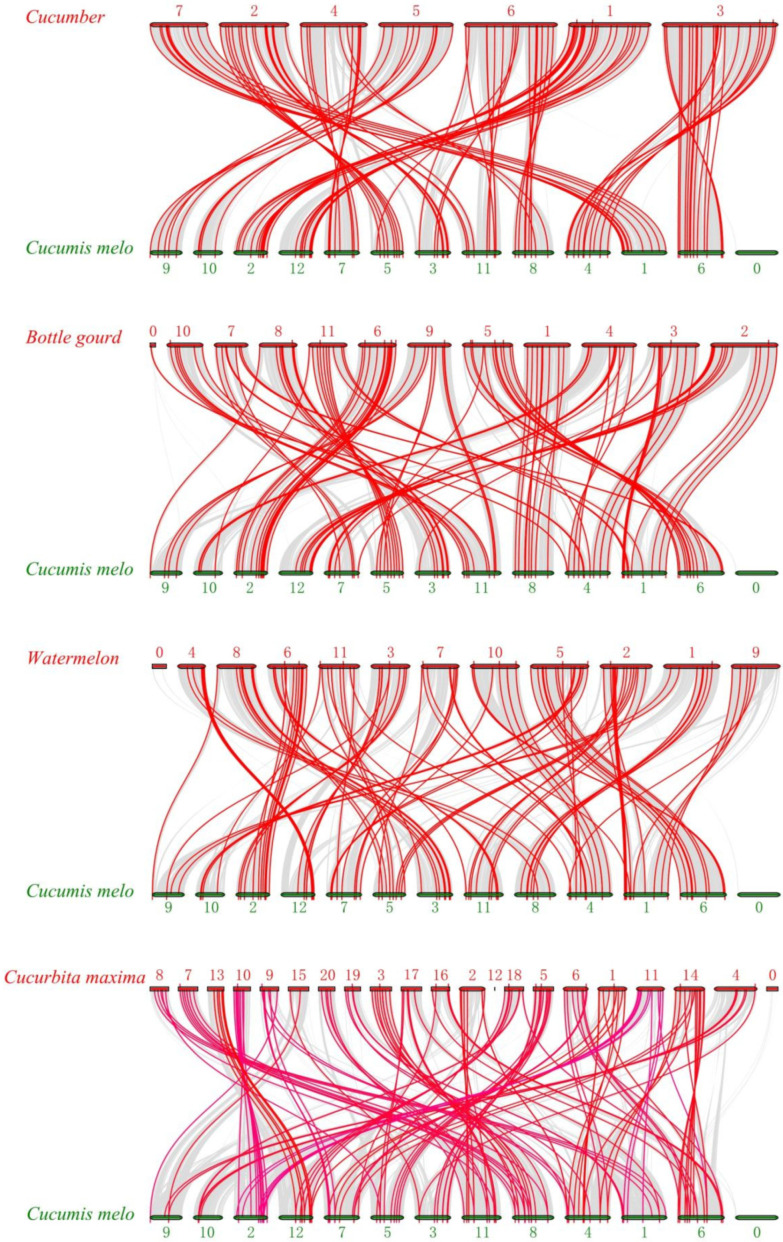
Collinearity analysis of *CmbHLHs* between melon and four Cucurbitaceae plants. The grey lines indicate the collinear blocks within melon and other plant genomes, and the numbers represent the chromosome numbers of plant genomes.

To further infer the origin and phylogenetic relationships of bHLH genes, comparative collinearity analysis between *Cucumis melo* and other cucurbit species was conducted. Figure 6 displays the collinearity relationship of *CmbHLH* genes with those in bottle gourd, cucumber, watermelon and *Cucurbita maxima* (Rimu) (Figure 6). A total of 115 *CmbHLH* genes were orthologous in the four species, among which 95 *CmbHLH* genes were common in the four species. Interestingly, 43 *CmbHLH* genes have at least two orthologues in Rimu, and these genes spread out on the 12 chromosomes of melon. The reason may be that the Rimu genome underwent a whole-genome duplication (WGD) event, which was not observed in the other four cucurbits (cucumber, melon, watermelon, and bitter gourd) [29]. Gene duplication events of *CmbHLH* in melon were also studied. The results showed 38 *CmbHLH* genes duplicated among *CmbHLH* genes (Figure S1). Except chromosome 9, all the other chromosomes have duplication genes of *CmbHLH*. Most of the duplicated genes were on chromosome 2, and 1 *CmbHLH* was locates on chromosome 6, illustrating an uneven distribution of the duplicated genes.

### 2.4. Expression Pattern of CmbHLH during Melon Fruit Development

**Figure 7:**
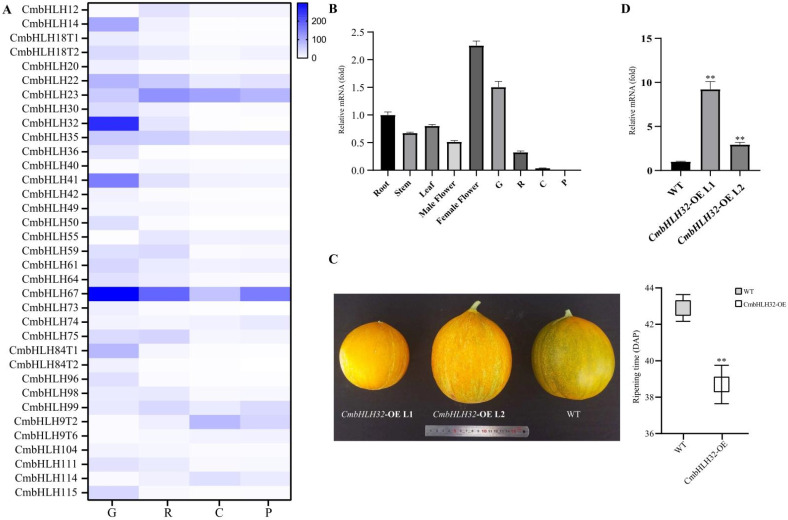
The differential expression profile of *CmbHLH*, different tissues and *CmbHLH32*-OE transgenic lines expression of *CmbHLH32*. (**A**) Heatmap of *CmbHLH* differential expression genes. (**B**) Quantitative PCR analysis of transcription levels of the *CmbHLH32* gene in different tissues. The root was normalized to 1. (**C**) Fruit ripening of *CmbHLH32*-OE transgenic lines were earlier than that of the WT. (**D**) The relative expression level of *CmbHLH32*-OE transgenic lines and WT fruit. Quantitative PCR expression level was calculated by 2^−^^ΔΔCT^ method, and the results are represented as the means ± standard deviations, ** *p* ˂ 0.01, N > 3.

Analysis of the expression data previously published from our laboratory of PRJNA543288 revealed 161 transcripts of 98 *CmbHLH* genes effectively expressed (i.e., in two replicate libraries) in melon fruit Growth stage (G), Ripening stage (R), Climacteric stage (C), and Post-climacteric stage (P) samples. However, most of them were weakly expressed; 45 *CmbHLH* genes had an average expression higher than 10 FPKM, and only 5 genes (*CmbHLH23*, *CmbHLH32*, *CmbHLH41*, *CmbHLH67* and *CmbHLH79*) had an average expression higher than 100 FPKM (Figure 7A). Differential expression analysis shows 32 *CmbHLH* genes differentially expressed in G vs. R, R vs. C and C vs. P stage samples. A total of 21 *CmbHLH* genes differentially expressed in G vs. R samples (7 genes upregulated and 14 genes downregulated), *CmbHLH32* was the highest expressed among these differential expression genes; 26 *CmbHLH* genes differentially expressed in R vs. C samples, only 2 genes (*CmbHLH9* and *CmbHLH114*) were upregulated, the others were downregulated. Six *CmbHLH* genes were differentially expressed in C vs. P stage samples. Taken together, the expression of *CmbHLH* genes exhibits a downregulation trend from melon fruit G to P stage samples, suggesting that most of the *CmbHLH* genes may function in the early fruit developmental stage. Whereas, two genes (*CmbHLH9* and *CmbHLH114*) were upregulated in R vs. C stage samples, indicating they may be involved in the regulation of fruit Climacteric.

### 2.5. Overexpression of CmbHLH32 Leading to Early Ripen in Melon Fruit

To further investigate the function of *CmbHLH* genes in fruit ripening, we generated *CmbHLH32* overexpression transgenic plant lines (CmbHLH32-OE). The reasons for studying *CmbHLH32* gene were: first, *CmbHLH32* was one of the highest expressed *CmbHLH* genes; second, *CmbHLH32* was the highest expressed among differential expression genes in G vs. R stage samples; third, tissue expression analysis results showed that *CmbHLH32* was highly expressed in female flower and early developmental stage fruit (Figure 7B); fourth, *CmbHLH32* was identified homolog to *AtbHLH93*, which has been proved to control *Arabidopsis* flowering by repressing *MAF5* [30]. However, blast analysis failed to find a homologous gene of *MAF5* in melon, and *CmbHLH32* also contains an ACT domain which is not present in *AtbHLH93*, suggesting that *CmbHLH32* may have a different function in melon flowering.

**Figure 8:**
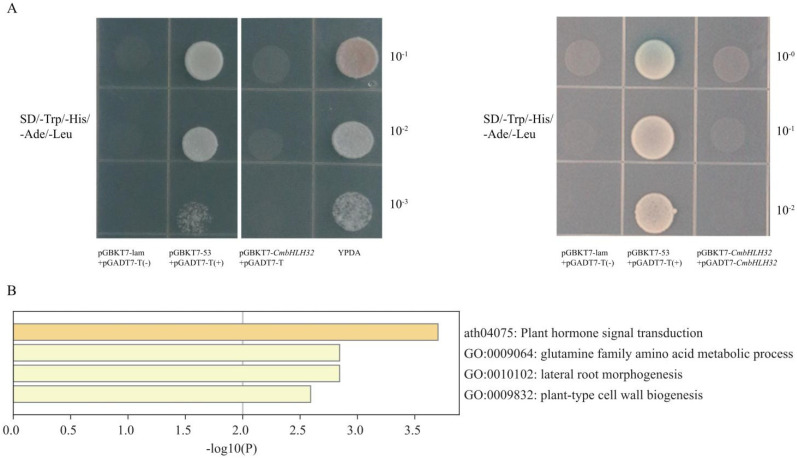
Transcriptional activation activity of CmbHLH32 and GO annotation of correlation expression genes of *CmbHLH32*. (**A**) Transcriptional activation activity of CmbHLH32. Full-length of *CmbHLH32* was fused to the GAL4 domain in the pGADT7-T vector and cotransformed with the pGBKT7-53 vector for monomer transcriptional activation activity of CmbHLH32. For homodimerization transcriptional activation activity tests, Full-length of CmbHLH32 fused to pGADT7 and pGBKT7 was cotransformed into the yeast AH109 strain. The growth performances of the transformed yeast cells were exhibited in different dilutions on SD/-Trp/-His/Ade/3-AT solid medium. (**B**) GO annotation of correlation expression genes of CmbHLH32 genes.

Transgenic T1 seeds overexpressing *CmbHLH32* were generated by the ovary injection method. Fruit ripening-related phenotype observation of CmbHLH32-OE T1 plants that were PCR detection positive indicated that overexpression of *CmbHLH32* resulted in early fruit ripening compared to the wild type (WT) melon fruit (Figure 7C). The fruit ripening of CmbHLH32-OE line (approximately 38.7 ± 1.1 DAP) was on average 4 days earlier than that of the WT (approximately 42.6 ± 0.8 DAP). Quantitative RT-PCR analysis showed that the expression level of the *CmbHLH32* gene increased an average of about 5.5 times campared with WT fruit (Figure 7D). Transcriptional activity of *CmbHLH32* was also studied by yeast two-hybrid assays; however, neither monomer nor homodimerization of *CmbHLH32* showed transcriptional activation activity in yeast (Figure 8A).

Analysis of fruit weight, length, width and fruit soluble solids content showed no difference between WT melon fruit and CmbHLH32-OE transgenic line fruits. However, CmbHLH32-OE transgenic plant fruits exhibit less firmness than WT melon fruits (Table 1). Expression correlation network analysis using the transcriptome data suggested 94 genes correlated with CmbHLH32 (Table S2). Further Gene Ontology (GO) analysis of correlated genes revealed that the GO term plant-type cell wall biogenesis (GO:0009832) was enriched (Figure 8B), suggesting that CmbHLH32 may affect fruit softening by regulating plant cell wall synthesis.

## 3. Discussion

### 3.1. Characteristics of CmbHLH Genes in Melon

Although the bHLH family has been widely studied in a diverse range of plants, the present study is the first to report the identification and characterization of bHLH transcription factors based on the entire genome sequence of *Cucumis melo*. In this study, 118 CmbHLH genes were identified and classified into 16 subfamilies and 4 orphan genes. Previous studies have revealed that most of bHLH proteins contained 19 conserved amino acid residues, for example in *Arabidopsis* and Chinese cabbage [6,27]. However, melon CmbHLH proteins contained 24 conserved amino acid residues, which was similar to the tomato bHLH proteins. There were more conserved amino acids in the second helix motif in the CmbHLH and SlbHLH proteins.

Proteins containing the HLH motif often form homo or heterodimers with other bHLH proteins. Leu-23 and Leu-52 (Leu-60 in melon) residues of helix 1 and 2, respectively, are structurally necessary for dimer formation of plant bHLHs and have been identified as the most conserved residues across plant bHLHs [11,31]. Interestingly, five melon CmbHLHs have mutation in Leu-23 or Leu-60 or both. Leu-23 was mutated to Phe-23 and Ile-23 in CmbHLH31 and CmbHLH58, respectively; Leu-60 mutant to Met-60 in CmbHLH57 and CmbHLH91; and in CmbHLH62, Leu-23 and Leu-60 was mutated to Val-23 and Glu-60, respectively. In *Arabidopsis*, mutation in the two Leu sites significantly affects the dimerization of the bHLH [32]. This finding indicates that a different functional mechanism may exist for at least on these five CmbHLHs.

Domain analysis of the CmbHLHs illustrated that eight proteins possessed an ACT domain. ACT domain is a small regulatory domain involved in amino acid or purine metabolism and can bind to varieties of ligands [33]. Mas-Droux et al. (2006) found a Lys and S-adenosylmethionine–sensitive Asp kinase isoform form a dimeric structure through ACT domain in *Arabidopsis* [34]. Kong et al. (2012) revealed that bHLH DNA-binding activity is suppressed if the C-terminal ACT domain is licensed to homodimer, and this protein-protein interaction domain is important for the regulation of anthocyanin pigment biosynthesis in maize [35]. Eleven MYC type CmbHLH were found. In plants, MYC genes participate in growth and development, and are also key regulators of the jasmonic acid (JA) signaling pathway [36,37]. This result indicates CmbHLHs may be involved in a more complex regulatory network.

### 3.2. Potential Roles of CmbHLH Genes in Melon Fruit Developmen

In the past few decades, characterization and functional analysis of the bHLH family in several species have been widely and extensively investigated. As one of the largest transcription factor families, bHLH functions in the regulation of plant growth and development, abiotic/biotic stress response, hormone signaling, iron homeostasis, and secondary metabolism. For example, in *Arabidopsis*, brassinosteroid (BR) and gibberellin can promote cell elongation by inhibiting an atypical the bHLH transcription factor INCREASED LEAF INCLINATION1 BINDING bHLH1 (IBH1), and ectopic accumulation of IBH1 causes a dwarf phenotype in *Arabidopsis* [38]. In soybean, the *GmORG3-like* gene enhances cadmium tolerance by increasing iron and reducing cadmium uptake and transport from roots to shoots [39]. However, studies on bHLHs regulating fruit growth and development are less investigated. Waseem et al. (2019) found that overexpression of *SlbHLH22* led to earlier fruit ripening and production of more ethylene-producing phenotypes in tomato [25]. Zhao et al. used white-flesh mutant strawberry to identify seven *FabHLH* genes that are responsive to the fruit anthocyanin biosynthesis and hormone signal transduction [40].

Using the transcriptome data, we studied the expression of *CmbHLH* during different fruit developmental stages. Three *CmbHLH* (*CmbHLH14*, *CmbHLH32* and *CmbHLH41*) genes were highly expressed (>100 FPKM) in the G stage and downregulated in the R stage. CmbHLH14 shares 74% identity with UNE12 of *Arabidopsis*, which might be involved in the regulation of the specific processes required for fertilization, and as a temperature-responsive SA immunity regulator [41,42]. CmbHLH23 was upregulated in the R stage, and was a homologous gene of the AtbHLH68, which was proposed to regulate lateral root elongation, and in the response to drought stress, it is likely to go through an ABA-dependent pathway in Arabidopsis [43]. CmbHLH59 showed significant downregulation in R vs. C stage samples. CmbHLH59 is an MYC-type protein that is differentially expressed from developmental to ripening stages in melon fruit. The CmbHLH59 sequence is similar to ATbHLH13 in *Arabidopsis*, and the functions of ATbHLH13 are repressing *Arabidopsis* defense responses and regulating anthocyanin biosynthesis through JA signaling pathway [44,45]. *CmbHLH114* was upregulated in R vs. C stage samples, and *CmbHLH114* was annotated to an ICE1-like gene. In *Arabidopsis*, *ICE1* is a multifunctional gene that responds to cold stress, ABA signaling regulation, determination of seed dormancy and so on [46,47]. In banana, MaICE1 targets to MaNAC1 during fruit storage in the cold and enhances fruit cold tolerance [48]. The expression of *CmbHLH67* was downregulated in the R vs. C stage and upregulated in the C vs. P stage. CmbHLH67 shares 59% identity with AtMYC2 of Arabidopsis, and responds to a variety of JA-dependent functions including secondary metabolism, insect resistance and stress tolerance [49,50]. In apple fruit ripening, MdMYC2 is required for JA-induced ethylene biosynthesis, and MdMYC2 was also found to interact with MdERF2, which is a downstream molecule of ethylene signaling [51]. The mature-fruit abscission of fleshy fruit is an obvious characteristic of fruit ripening. By analyzing the transcriptome data of melon abscission zone (AZ), Corbacho et al. found some genes that may regulate fruit abscission. Some *bHLH* genes were found to be involved in regulating the ripening and abscission of melon fruit. *bHLH44* is not differentially expressed in our transcriptome data, and its FPKM is only 70–90. Therefore, whether it regulates the ripening of fruit needs further study [52].

In this study, we observed that overexpressing *CmbHLH32* led to early fruit ripening. This result suggests that *CmbHLH32* may participate in the growth and ripening of melon fruit, however, a detailed study is still needed. In apple, leaf senescence and the expression of senescence-related genes were promoted by MdbHLH93 (homolog to *AtbHLH93*), and leaf senescence was delayed when an ABA-responsive protein, MdBT2, interacted directly with MdbHLH93 [53]. In *Arabidopsis*, double mutants of *bHLH93* failed to flower under short day (SD) conditions and *bHLH93* plays a major role in regulating *Arabidopsis* SD flowering [30]. Tissue expression analysis illustrates a high expression level of *CmbHLH32* in female flower, suggesting *CmbHLH32* may play a regulatory role in flowering. However, melon is a day-neutral plant, suggesting *CmbHLH32* may have a different function than the *bHLH93* in *Arabidopsis*. Yeast tw-hybrid analysis indicates that the transcriptional activation activity of CmbHLH32 may depend on other proteins. Melon CmbHLH32 contains an ACT domain, and ACT domain was first identified as a ligand-binding domain, which was found to suppress bHLH DNA-binding activity [35,54]. This could explain why the homodimer of CmbHLH32 did not show transcriptional activation activity in vitro.

## 4. Methods and Materials

### 4.1. Sequence Retrieval and Identification of bHLH Proteins in Melon

The Hidden Markov Model method and blast method were used to identify the melon bHLH protein sequences. Melon protein sequences (CM3.5.1_protein) were downloaded from the Cucurbit Genomics Database (CuGenDB, http://cucurbitgenomics.org/, accessed on 3 May 2019) [55]. The protein sequences of *A. thaliana* bHLH (AtbHLH, Araport11_genes. 201606.pep.fasta) were retrieved from The Arabidopsis Information Resource (TAIR), (https://www.arabidopsis.org/download/index-auto.jsp?dir=/download_files/Proteins, accessed on 3 May 2019), on www.arabidopsis.org(accessed on 3 May 2019). The Hidden Markov Model (HMM) file of the HLH domain (PF00010) was downloaded from the Pfam database (version 32.0; http://pfam.xfam.org/, accessed on 3 May 2019) [56]. The HMM software (version3.2.1; http://www.hmmer.org/, accessed on 3 May 2019) was used to search against the melon protein sequence data using default parameters [57]. The *Arabidopsis* bHLH protein sequences were used to blast against the melon protein sequences using Blast software by default parameters [58]. Then sequences were compared to the CmbHLH proteins downloaded from PlantTFDB (http://planttfdb_v4.cbi.pku.edu.cn/, accessed on 3 May 2019), and repeated sequences were removed. After that these protein sequences were submitted to the online Batch CD-search tool (https://www.ncbi.nlm.nih.gov/Structure/bwrpsb/bwrpsb.cgi, accessed on 4 May 2019) to verify the existence and integrity of the conserved bHLH domain [59,60]. The sequences above the minimum threshold bit score were kept, and the redundant sequences were removed. These representative sequences of putative *CmbHLHs* were named based on their chromosome location. Transcriptome data using previously published data from our laboratory (PRJNA543288).

### 4.2. Identification of Conserved Motifs and Tandem Repeat Genes

The molecular weight (Mw) and theoretical isoelectric point (pI)-values for these CmbHLH protein sequences were determined by the Compute pI/Mw tool on the ExPASy server (http://web.expasy.org/compute_pi/, accessed on 1 June 2019) [61]. Conserved motifs of CmbHLHs were identified by MEME (http://meme-suite.org/, accessed on 1 June 2019) server with maximum number of motifs set at 10 and optimum width of motifs from 6 to 70 amino acids [62]. MCscanX software was used to identify the tandem repeat genes of the *CmbHLHs* using the default parameters. Additionally, the results were displayed by the TBtools software (version 0.66682) [63,64].

### 4.3. Gene Structure and Collinearity Analysis

Gene structure (intron-exon) information of these putative *CmbHLHs* was obtained from the GFF (CuGenDB, CM3.5.1_pseudomol_gene_model.gff) file and displayed by the TBtools software. Intron distribution pattern within bHLH domain of CmbHLHs was analyzed manually. The cds sequences and gff file of cucumber (Chinese long v2), watermelon (Charleston Gray v2), *cucurbita maxima* (Rimu v1.1) and Bottle gourd (*Lagenaria siceraria* cv. USVL1VR-Ls v1) were downed from CuGenDB [29,65,66,67]. Collinearity analysis of the four species and melon was performed by the MCscanX software(version 1).

### 4.4. Phylogenetic Analyses of CmbHLHs

Multiple alignment of the putative melon CmbHLHs proteins were used MEGA X software (version 10.0.5) by MUSCLE program with default options, the maximum likelihood (ML) method were used to construct phylogenetic trees by PhyML 3.0 software, with VT (variable time) amino acid substitution model and 1000 replications of bootstrap values [68,69].

### 4.5. Expression Analysis and GO Annotation

Expression value (FPKM) of CmbHLHs transcripts were extracted from the transcriptome expression data sets, and genes expression correlation network were constructed by Expression correlation plugin (version 1.1.0) in Cytoscape software (version 3.7.1) [70], genes with correlation coefficient <−0.99 or >0.99 and E-box (CANNTG) sequence in their promoter sequence (1000 bp) were kept for later analysis. GO annotation were conducted by online website Metascape (http://metascape.org/gp/index.htm, accessed on 17 July 2019) [71].

### 4.6. Plant Material and Growth Condition

Melon (*Cucumis melo* cv. Hetao) plants were grown in green house at Dengkou (40°19′46.07″ N, 107°0′11.46″ E), Inner Mongolia Autonomous Region, China. Hetao melon is a conventional melon variety planted in the western part of Inner Mongolia. It has been cultivated for more than 70 years in the local area. The fruit of Hetao melon is respiratory climacteric fruit, small fruit and medium early maturing variety, which can be cultivated in field or in greenhouse. The melon plants that we used to construct transgenic plant were using the above-mentioned cultivars, after our laboratory 11 generations of self-pollination breeding selection and obtained typical and stable inbred lines of Hetao melon. The flowers were self-pollinated, the pollination time was recorded, and the date of pollination was recorded as 0 days after pollination (DAP). Only one fruit was kept for each plant. We defined the ripening time of Hetao melon as when the peel of the melon just turned full yellow and the fruit stalk just began to form an abscission zone (AZ) [72].

The wild-type melon seeds, which were from the above mentioned stable inbred lines of Hetao melon, sterilized by mercuric chloride were planted in 1/2 MS medium at 25 °C, light (1000 lx) for 16 h, dark treatment for 8 h, and cultured for 30 days to obtain the root, stem and leaf tissues of melon. The male and female flowers of wild type melon without pollination which were planted in greenhouse were collected and preserved immediately in liquid nitrogen.

### 4.7. Gene Cloning and Transgenic Plant Generation

An open reading frame of 1023bp of *CmbHLH32* (*MELO3C011110*) gene was amplified by RT-PCR from melon total RNA using upstream primer: 5′-atggagagctcacgaacgtctt-3′ with a *Kpn*I restriction site and downstream primer: 5′-ctacagcagcttcctcatac-3′ with a *Xba*I restriction site. The PCR products were cloned into the vector of pMD-19T (Takara Bio, Shiga, Japan) by TA cloning. Subsequently, the fragment was inserted into the overexpression vector pPZP221 applying *Kpn*I and *Bam* HI sites. The plasmid pPZP221-CmbHLH32 was diluted with 2 × SSC (pH 7.0) solution to 100 ng/μL. After 7 h of artificial pollination, 100 ng/μL of plasmid solution was injected into the pistil ovary by ovary injection method to obtain transgenic plant.

T1 transgenic plants were identified by PCR test using two primer sets, one is CaMV35S upstream primer: 5′-CAGAAAGAATGCTAACCC-3′ and downstream primer: 5′-TTCTTCTTGTCATTGAGTCGTA-3′; another is upstream primer: 5′-TTTCGGTCGTGAGTTCGGAG-3′ and downstream primer: 5′-CACTTCTTCCCGTATGCCCA-3′. Only if the plant that was detected by both of these primers will be preserved for later observations.

### 4.8. Quantitative Real-Time PCR Analysis

A quantitative real-time PCR (qRT-PCR) assay was performed to validate the expression of CmbHLHs. Total mRNAs were reverse transcribed using PrimeScript™ RT reagent Kit with gDNA Eraser kit (Takara Bio, Shiga, Japan) following the manufacturer. The qRT-PCRs were performed using SYBR^®^ Premix Ex Taq™ II (Takara Bio, Shiga, Japan) by a 96-well Chromo4 Real-Time PCR system. The qRT-PCR conditions were as follows: a predenaturation of 30 s at 95 °C, followed by 35 cycles of 5 sec at 95 °C and 30 sec at 60 °C. The 2^−ΔΔCT^ method was used to analyze the relative mRNA expression level. The control gene was *CmGAPDH* (glyceraldehyde-3-phosphate dehydrogenase).

### 4.9. Transcriptional Activation Activity Analysis in Yeast

Full length of *CmbHLH32* was cloned and inserted into pGBKT7 and pGADT7 vectors (TaKaRa) to obtain pGBKT7-CmbHLH32 and pGADT7- CmbHLH32 vectors. The pGBKT7-CmbHLH32 was transformed into the yeast strain AH109 (TaKaRa) with pGADT7-T vector for transcriptional activation activity test. Homodimerizing transcriptional activation activity of *CmbHLH32* used cotransformation of pGBKT7-CmbHLH32 and pGADT7- CmbHLH32 vectors. Cotransformation of pGBKT7-53 + pGADT7-T and pGBKT7-Lam + pGADT7-T were used as positive control and negative control respectively. The transformed yeast cells were streaked on SD/-Trp/-His/Ade/3-AT solid medium in different dilutions (10^−1^, 10^−2^ and 10^−3^). After 3-day incubation at 30 ℃, the transcriptional activation activity of *CmbHLH32* were evaluated according to growth status of transformed yeast cells.

## 5. Conclusions

This study was focused on the identification of the melon *bHLH* gene family and the function of *bHLH* genes in fruit growth and ripening. We identified the *CmbHLH* gene family in melon and demonstrated the CmbHLH classification, the bHLH domain characteristics and intron patterns, and phylogenetic relations of CmbHLH and four other cucurbit species. Expression characteristics of *CmbHLH* genes using the transcriptome data suggested that most of these *CmbHLH* genes were expressed at low/no lexels during fruit development. Furthermore, *CmbHLH* genes tended to be expressed in early stage of fruit development, among which *CmbHLH32* was the most prominent. Expression of *CmbHLH32* was high in female flowers and early fruit growth stage. Transgenic plant lines overexpressing *CmbHLH32* result in early fruit ripening compared to the wild type fruits. These findings clarify the members and characteristics of the *CmbHLH* gene family, and provide new insights into the role of melon bHLH genes in regulating fruit development.

## Figures and Tables

**Table 1:** Phenotypes of wild type (WT) and *CmbHLH32*-overexpressing (*CmbHLH32-OE*) transgenic melon plants.

Parameter	WT	*CmbHLH32*-OE L1	*CmbHLH32*-OE L2
fruit weight (g)	961.8 ± 155.3	954.1 ± 169.4	890.5 ± 113.1
Hrizontal diameters of fruit (cm)	12.7 ± 0.9	12.7 ± 0.9	12.6 ± 0.7
Vertical diameters of fruit (cm)	13.6 ± 1.0	13.6 ± 0.7	13.1 ± 0.7
soluble solids content (%)	13.7 ± 1.0	12.7 ± 2.9	11.6 ± 1.9
firmness of fruit (Kg)	5.5 ± 0.6	4.5 ± 0.4 (*)	4.0 ± 0.2 (*)

Values are means of 3–10 plants, ±SE. The statistical significance of mean differences was analyzed using Student’s *t*-test, *: *p* < 0.05(compared with WT).

## Data Availability

The datasets analyzed during the current study are available in the SRA (https://www.ncbi.nlm.nih.gov/sra, accessed on 1 June 2019), SRA accession: PRJNA543288.

## Supplementary Materials

Supplementary materials can be found at https://www.mdpi.com/article/10.3390/plants10122721/s1. Figure S1. Gene duplication of melon CmbHLH. Red lines links the duplicate genes of CmbHLHs, grey lines are background of other genes duplication. Chromosomes are indicated by light yellow bars. (PDF 457 kb), Table S1. Sequences, gene ID and other information of melon *CmbHLH*. (xlsx 40 kb), Table S2. Correlation genes of *CmbHLH32.*

## Abbreviations

bHLH: Basic helix–loop–helix; TF: Transcription factor; DAP: days after pollination; SFT: SINGLE FLOWER TRUESS; LFY: LEAFY; ACC: 1-Aminocyclopropane-1-carboxylate; IAA: auxin indole-3-acetic acid. MAF5: FLOWERING LOCUS M (FLM)/MADS AFFECTING 5.
