# Supplementary material for: Genome-Wide Identification and Characterization of Melon bHLH Transcription Factors in Regulation of Fruit Development
Source: Plants (Basel). 2021 Dec 10;10(12):2721. doi: 10.3390/plants10122721 (PMC8709311; doi:10.3390/plants10122721)
Supplement: Supplementary file 1 [file plants-10-02721-s001.zip › Supplementary Figure S1.pdf]

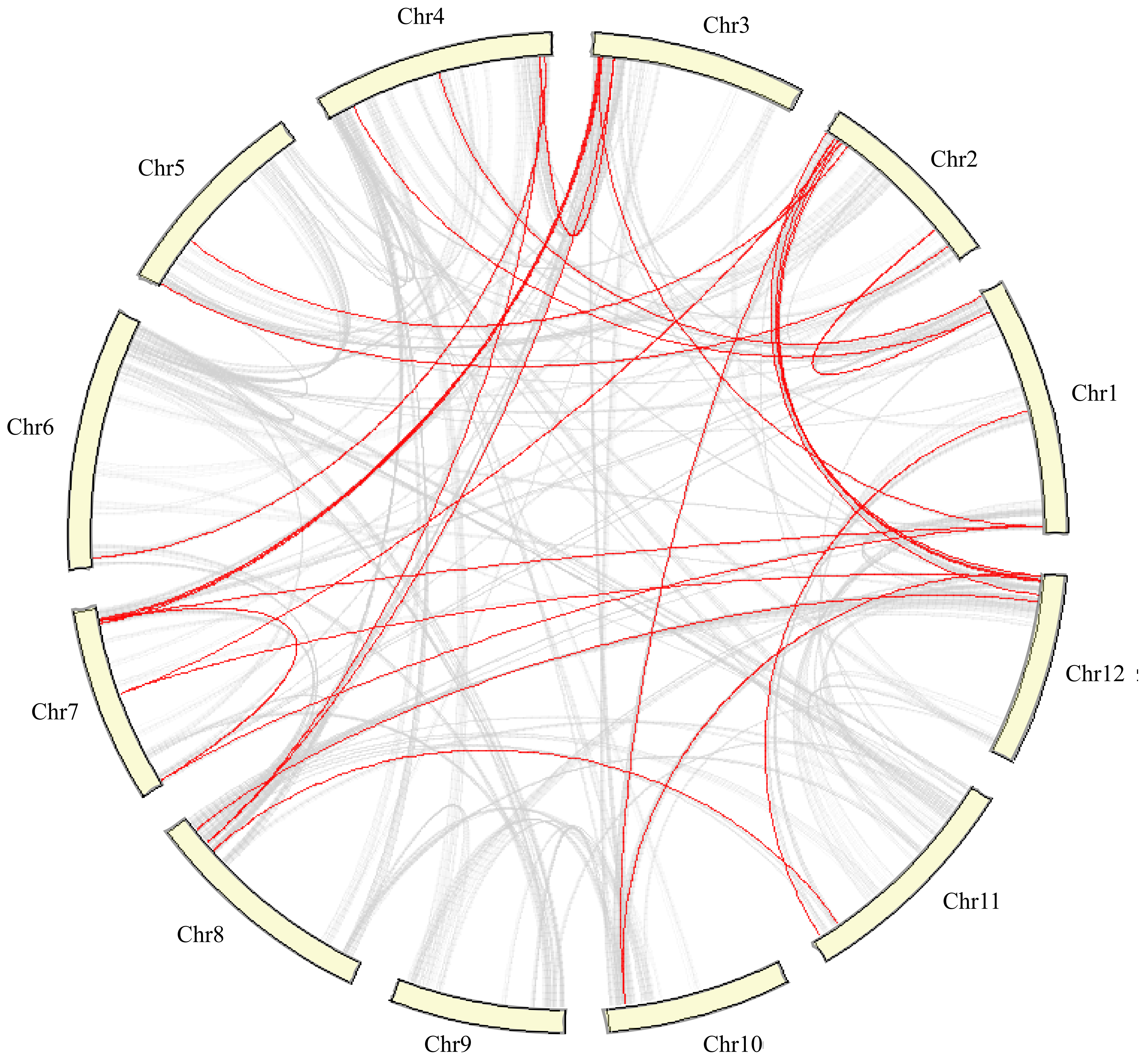

Supplementary Figure S1 Gene duplication of melon CmbHLH. Red lines links the duplicate genes of CmbHLH, grey lines are background of other genes duplication. Chromosomes are indicated by light yellow bars.
